# Aneuploidy Detection in Pigs Using Comparative Genomic Hybridization: From the Oocytes to Blastocysts

**DOI:** 10.1371/journal.pone.0030335

**Published:** 2012-01-23

**Authors:** Miroslav Hornak, Eva Oracova, Pavlina Hulinska, Leona Urbankova, Jiri Rubes

**Affiliations:** 1 Veterinary Research Institute, Brno, Czech Republic; 2 Institute of Animal Science, Kostelec nad Orlici, Czech Republic; Karolinska Institutet, Sweden

## Abstract

Data on the frequency of aneuploidy in farm animals are lacking and there is the need for a reliable technique which is capable of detecting all chromosomes simultaneously in a single cell. With the employment of comparative genomic hybridization coupled with the whole genome amplification technique, this study brings new information regarding the aneuploidy of individual chromosomes in pigs. Focus is directed on *in vivo* porcine blastocysts and late morulas, 4.7% of which were found to carry chromosomal abnormality. Further, ploidy abnormalities were examined using FISH in a sample of porcine embryos. True polyploidy was relatively rare (1.6%), whilst mixoploidy was presented in 46.8% of embryos, however it was restricted to only a small number of cells per embryo. The combined data indicates that aneuploidy is not a prevalent cause of embryo mortality in pigs.

## Introduction

Chromosomal abnormalities presented in embryos are a major cause of pregnancy loss, largely impair correct embryo and foetus development or lead to the birth of individuals suffering from various congenital abnormalities.

Compared with humans, the data on incidences and the nature of chromosomal abnormalities in farm animals are much more limited because there is no such rigorous monitoring of embryo/foetus development during the prenatal period and the samples of miscarriages or abnormal animals are rarely sent to cytogenetic laboratories for examination. Nevertheless, numerical errors such as trisomy of particular chromosomes, monosomy of chromosome X, polyploidy, as well as structural chromosome abnormalities encompassing reciprocal and Robertsonian translocations, inversions or insertions exist in farm animals [1], which closely resemble the abnormalities commonly found in humans. However, the incidence and character of chromosome abnormalities differ in gametes or embryos of individual animal species [1]–[5]. An example might be a relatively high incidence of reciprocal translocations found in pigs [6]. Furthermore, the literature shows the frequency of aneuploidy in oocytes or embryos vary, even in the same species and are likely affected by different circumstances, e.g. by the different age of animals used for experiments, methods employed or by the *in vitro* cultivation processes compared to *in vivo* samples. In Table 1, we have summarized the most relevant publications concerning pig aneuploidy emphasizing the abovementioned significant factors.

**Table 1:** Published frequency of aneuploidy in pigs.

Study Details				Frequency of Errors			Reference
Sample	Conditions	Sample donors	Method	Chromosome	Ploidy		
				Errors (%)	Errors (%)		
Sperm	in vivo	Boar donor	FISH	∼0.3^a^	∼0.2	-	[15]
Oocytes	in vivo	1^st^ estrous gilts	Chromosome spreads	10.8^c^	-	-	[29]
		3^rd^ estrous gilts	Chromosome spreads	5.9^c^	-	-	
Oocytes	in vitro	Cycling gilts	FISH	3.0^b^	-	-	[14]
Oocytes	in vitro	Cycling gilts	Chromosome spreads	4.9^c^	-	-	[16]
Oocytes	in vitro	Prepubertal gilts	FISH	10.8^b^	-	-	[12]
		Aged sows		1.3^b^	-	-	
Oocytes	in vitro	Miniature and crossbreed cycling gilts	CGH	∼10.0	-	-	[7]
Early embryos	in vivo	Cycling gilts	Chromosome spreads	-	**1.2** d	7.3^e^	[23]
Early embryos	in vivo	Crossbreed cycling gilts	FISH	1.8^b^	**1.8** d	9.6^e^	[13]
Early embryos	in vivo	Crossbreed cycling gilts	CGH	14.3	-	-	[8]
Day 6 blastocysts	in vivo	Crossbreed cycling gilts	CGH+FISH	4.7	**1.6** d	48.4^e^	present study
Day 6 blastocysts	in vivo	Cycling sows	FISH	-	**0.0** d	75.0^e^	[21]
	in vitro	Cycling sows	FISH	-	**0.0** d	95.0^e^	
Day 6 blastocysts	in vitro	Prepubertal gilts	Chromosome spreads	-	**31.4** d	39.1^e^	[20]
Day 6 blastocysts	in vitro	Prepubertal gilts	Chromosome spreads	-	**23.4** d	45.9^e^	[30]
Day 10 blastocysts	in vivo	Large White sows	Chromosome spreads	-	**5.1** d	64.5^e^	[24]
Day 10 blastocysts	in vivo	Crossbreed cycling gilts	Chromosome spreads	-	**-**	10.0^e^	[25]
Day 10 blastocysts	in vivo	Crossbreed sows	Chromosome spreads	-	**6.7** d	6.7^e^	[26]
Day 10 blastocysts	in vivo	Prepubertal gilts	Chromosome spreads	-	**0.0** d	-	[22]

a only chromosomes 1, 10 and Y detected.

b only chromosomes 1 and 10 detected.

c only hyperhaploidy.

d only true polyploidy without mixoploidy.

e polyploidy+mixoploidy.

A need for reliable technique, capable of obtaining the maximum information from an examined sample of animal oocytes or embryos, is required. Recently, we have utilized comparative genomic hybridization (CGH) coupled with whole genome amplification (WGA) in order to study porcine oocytes and early embryos [7], [8]. This protocol is routinely used in human pre-implantation genetic diagnosis and starts to replace well established FISH analysis for this purpose [9], [10]. As discussed later, the main advantage of the WGA-CGH approach over traditional techniques is the possibility to screen all chromosomes in a single cell. On the other hand, the main drawback represents an inability to detect polyploidies.

We have reported the frequency of aneuploidy in porcine oocytes and in *in vivo* early embryos (collected 3 days after insemination) to be 10.1% and 14.3%, respectively [7], [8]. In our present study we focused on *in vivo* obtained porcine blastocysts (collected 5.5 days after insemination). Considering that CGH is not able to detect polyploidies, we extended our work and enumerated the incidence of polyploidy in *in vivo* porcine blastocysts using FISH. Obtaining information from porcine oocytes, early embryos [7], [8], and from the current study of porcine blastocysts using the novel WGA-CGH approach, we would like to detail to what extent is aneuploidy the cause of embryo mortality in animals, particularly in pigs.

## Results

**Figure 1:**
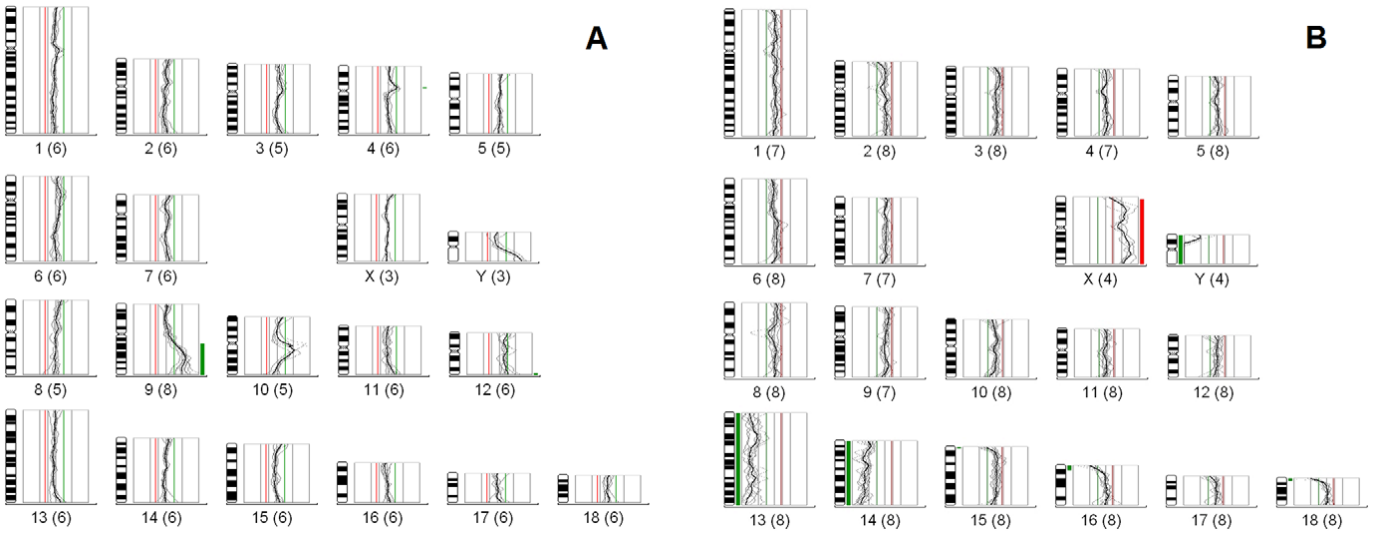
The example of WGA-CGH analysis of 2 aneuploid pig embryos. (A) the male embryo detected with partial loss of chromosome 9q; (B) the female embryo detected with loss of chromosomes 13 and 14. Amplified DNA obtained from the embryo was labelled with red fluorescence and amplified reference male porcine DNA was labeled with green fluorescence. Both DNA samples were mixed and allowed to hybridize to male porcine mitoses. Subsequently, the red and green fluorescence was captured and analyzed using dedicated CGH software. The heterochromatin regions (e.g. centromeres and the q arm of chromosome Y) were excluded from the analysis due to the abundance of repetitive DNA sequences.

In total, 90 *in vivo* derived pig embryos from 10 cycling gilts were isolated to study abnormalities of an entire chromosome set using WGA-CGH. Eighty-five embryos were at blastocyst stage and 5 embryos were at late morula stage, however were included into the analysis due to a higher number of cells (>32 cells). Eighty-six of the 90 embryos (96%) were successfully examined, 3 embryos did not amplify (probably due to loss of embryos during the transfer into PCR tube) and 1 embryo showed an uninterpretable CGH profile. The sex ratio was 0.95 (42 M∶ 44 F). Overall, 4 aneuploidies out of the 86 successfully examined embryos (4.7%) were detected, 3 embryos contained a loss of the whole chromosome(s) and 1 embryo contained a partial loss of the 9q chromosome. Data on the collection of embryos, aneuploidy, and sex are summarized in Table 2. An example of a WGA-CGH analysis of aneuploid embryos is shown in Figure 1.

**Table 2:** The incidence and description of aneuploidies in pig embryos detected by CGH.

Gilt No.	No. of Embryos					Aneuploidy Description	
	Collected	Analyzed	Normal	Aberrant	Sex Ratio M/F	Embryo No. 1	Embryo No. 2
1	12	11	10	1	3/8	XX,-13,-14^a^	
2	4	4	4	0	2/2		
3	10	9	8	1	3/6	XX,-13	
4	11	11	11	0	5/6		
5	9	9	7	2	6/3	XY,-14	XY,-9q
6	7	7	7	0	5/2		
7	9	8	8	0	4/4		
8	13	12	12	0	6/6		
9	9	9	9	0	5/4		
10	6	6	6	0	3/3		
**Total No.**	**90**	**86**	**82**	**4**	**42/44**		

a aneuploid embryo was at the late morula stage.

The table summarizes the numbers of embryos collected and analyzed from individual gilts. Besides that, data on the sex ratio, numbers of abnormal embryos and the description of chromosome abnormalities are provided.

Since CGH detects all chromosomes, we were able to determine individual chromosome contribution to aneuploidy, results are depicted in Table 3. In the present study of blastocysts, chromosome losses were the only aneuploidy finding. It is of interest, that the largest porcine acrocentric chromosomes (chr. 13 and 14) were both involved in aneuploidy twice.

**Table 3:** The occurrence of individual pig chromosomes in aneuploid oocytes, early embryos and blastocysts.

CGH analysis		Individual Chromosomes																			
Type of Sample	No. of AneuploidSamples	1	2	3	4	5	6	7	8	9	10	11	12	13	14	15	16	17	18	X	Y
**Porcine Oocytes** a	**13**	1	2	0	0	1	1	0	2	1	0	0	2	3	1	3	0	0	0	1	n/a
**Early Porcine Embryos** b	**8**	2	0	1	0	0	0	0	2	0	0	1	2	1	1	1	1	1	0	2	1
**Porcine Blastocysts** c	**4**	0	0	0	0	0	0	0	0	0	0	0	0	2	2	0	0	0	0	0	0
**Total No.**	**25**	**3**	**2**	**1**	**0**	**1**	**1**	**0**	**4**	**1**	**0**	**1**	**4**	**6**	**4**	**4**	**1**	**1**	**0**	**3**	**1**

a the results of the CGH analysis of oocytes were published in [7].

b the results of the CGH analysis of early embryos were published in [8].

c present study.

The table shows individual pig chromosomes and their occurrence in aneuploid samples. The aneuploid samples containing >3 chromosome abnormalities per oocyte/embryo (complex aneuploidy with a likely stochastic distribution of chromosome errors) and samples containing segmental chromosome abnormalities were excluded.

In order to detect ploidy errors (polyploidy, haploidy and mixoploidy), 62 out of 76 *in vivo* derived pig embryos (82%) were successfully fixed on the slide and examined using FISH. In total, 4412 nuclei were analyzed (71.1±26.0 per embryo). The remaining 14 embryos did not contain a minimum of 30 cells after fixation on the slide, and therefore were excluded from the analysis. Out of 62 examined embryos, 60 embryos were at blastocyst stage and 2 embryos were at late morula stage with higher number of cells (>32 cells). Only 1 blastocyst was triploid. Twenty-nine embryos were mixoploid (46.8%), however, only 6 embryos contained more than 5% of cells with ploidy abnormality. We have found that tetraploidy was the prevalent aneuploidy in mixoploid embryos. Comprehensive data on aneuploidy examined using FISH is shown in Table 4.

**Table 4:** The incidence and description of ploidy abnormalities in pig embryos detected by FISH.

Cells with Ploidy Abnormalities%	Embryos		Aneuploidy Description						
	No.	%	2n	2n	2n	2n	2n	2n	2n
			+	+	+	+	+	+	+
			1n	3n	4n	1n+3n	3n+4n	1n+4n	1n+3n+4n
0	32	51.6	-	-	-	-	-	-	-
0–5	23	37.1	4	4	11	1	1	2	-
6–10	4	6.5	1	-	-	1	1	1	-
11–15	-	-	-	-	-	-	-	-	-
16–20	1	1.6	-	-	1	-	-	-	-
21–30	1	1.6	-	-	-	-	-	-	1
31–40	-	-	-	-	-	-	-	-	-
41–50	-	-	-	-	-	-	-	-	-
51–99	1	1.6	-	1^a^	-	-	-	-	-
**Total No.**	**62**	**100**	5	4	12	2	2	3	1

a embryo contained 98% of triploid cells, therefore it is considered as triploid.

The frequencies of ploidy abnormalities are grouped with respect to the percentage of abnormal cells within individual embryos (first column). On the right side of the table, the numbers and description of ploidy mosaicism is given; for example, in the group of embryos with ploidy abnormalities 0–5%, 11 embryos contained beside diploid cells only tetraploid cells etc.

## Discussion

With the ability to detect all chromosomes in a single cell, the WGA-CGH represents a significant technological shift towards improved aneuploidy screening in oocytes or embryos. For example, using CGH technology on first polar bodies, it was newly observed that precocious separation of sister chromatids rather than non-disjunction of the whole bivalents is the predominant mechanism leading to aneuploidy in humans [11]. A FISH technique, which has been widely employed in farm animal aneuploidy research, generally only detects 2–3 chromosomes [12]–[15]. Given the fact, that the level of aneuploidy in animals is relatively low, the obtained data using FISH is likely to suffer from high statistical error, so a large group of samples is required to obtain unbiased data. Giemsa staining on chromosome spreads is also frequently performed in animal studies, however a chromosome spreading process is prone to various artifacts, e.g. poor quality, overlapping, loss of chromosomes [13], [14], [16]. This drawback is eliminated when using WGA-CGH since the examined single cell is placed intact in the PCR tube.

In the present study, we examined the embryo as a whole, hence the evaluation of mosaicism was not possible. Theoretically, if a particular chromosomal abnormality, e.g. monosomy or trisomy, was presented in 50% of embryo cells, the CGH ratio would be 0.75 and 1.25, respectively. The 0.75 and 1.25 was actually our threshold limit for chromosomal loss and gain, correspondingly, which implies that only a particular chromosomal aberration presented in the half of the embryo cells could be reliably detected. In other words, our experimentally observed frequency of aneuploidy in pig embryos is related to chromosome errors arising during meiosis or first divisions of the zygote, since such errors produce chromosome abnormalities in the majority of the embryo cells [17].

Probably, the most relevant drawback of WGA-CGH is its inability to detect polyploidies. To overcome this limitation, we have used a FISH method to assess polyploidy in *in vivo* pig embryos. The CGH experiments were conducted on Landrace and Czech Large White crossbreed pigs (LxCLW), but the FISH experiments were carried out on Prestice black pied pigs, because of a change in pig breed at the local farm. The true polyploidy observed in the blastocysts of Prestice black pied breed in the current study is almost identical (1.6% vs. 1.8%) compared to the true polyploidy frequency in the embryos of crossbreed pigs found in another study [8]. Therefore, we assume the polyploidy frequency in *in vivo* porcine embryos is similar in different breeds, however other studies focusing on the incidence of chromosomal abnormalities in different pig breeds are needed.

Using WGA-CGH, we found 4.7% (4/86) of blastocysts to be aneuploid and thus the frequency of aneuploidy is significantly lower (*p*<0.05) compared to early pig embryos, where the frequency was 14.3% (11/77) [8]. This observed difference in pigs confirms, that aneuploidy might be responsible for early embryo mortality or disturbed embryo development. Observing aneuploidy in 1 out of 5 late morulas compared to 3 out of 81 blastocysts further support this hypothesis. In regards to the character of aneuploidy, in blastocyst stage embryos, we found no complex aneuploidies (3 or more abnormal chromosomes). This indicates, that such abnormalities hamper embryo development and the majority of embryos with complex aneuploidy do not reach the blastocyst stage. In one blastocyst we found partial chromosome abnormality – loss of 9q. With the onset of CGH technology in human pre-implantation genetic diagnoses (PGD), partial chromosome errors have been commonly observed in cleavage stage embryos, but also in human blastocysts [18], [19]. Our findings confirmed that partial chromosome abnormalities also exist in pig embryos.

FISH provides accurate data on the incidence of polyploidy. In our study of *in vivo* derived blastocysts, only one out of 62 embryos was uniformly polyploid (triploid). A more common abnormality was mixoploidy (presented in 46.8% of embryos). However, it should be noted that the vast majority of mixoploid blastocysts consisted of only a few polyploid cells within the whole embryo. Moreover, solely tetraploid cells were observed besides diploid cells in some embryos. This can be explained by polyploidization of the trofectoderm, which naturally occurs in higher differentiated stages of embryos [20]. Finally, some ploidy abnormality might be attributed to the error rate of FISH method. Considering the aforementioned points, it would be more illustrative to apply 5% and 10% threshold of abnormal cells in mixoploid embryos. Only 9.7% (6/62) of embryos contained more than 5% of cells with ploidy abnormality and just 2 of them with more than 10% (in one embryo 16.2% and in second 27.3%) of abnormal cells (Table 4).

There are several studies concerning aneuploidy in pig oocytes and embryos. Several employed FISH technique to focus on only a few chromosomes and mathematically extrapolated data in order to estimate aneuploidy in the whole genome [12]–[14]. Another group of studies focused on ploidy abnormalities using FISH or chromosome spreading technique. Results of the most relevant studies are summarized in Table 1. The most striking finding was the high frequency of polyploidy in a group of *in vitro* derived pig blastocysts compared to those obtained *in vivo*. This suggests that pig might be quite sensitive to *in vitro* processes and as a result of these suboptimal artificial conditions the level of ploidy abnormalities rises significantly. Concerning mixoploidy, the obtained data vary greatly. From the methods used for aneuploidy screening, only the FISH technique was capable of examining all cells from individual embryos and thus bringing complex information on ploidy mosaicism. It was found that in *in vivo* pig blastocysts the ploidy mosaicism is restricted to only a minority of cells (approx. 5%) [21], and our present study supports that observation.

By employing WGA-CGH, we have provided novel data on the aneuploidy of individual chromosomes in porcine oocytes and embryos (Table 3). Our findings suggest that large acrocentric chromosomes (chromosome 13, 14 and 15) are often involved in aneuploid oocytes, early embryos and blastocysts and, surprisingly, we did not detect small chromosomes to be frequently aneuploid. This was concluded from 38 aneuploid chromosomes presented in 25 aneuploid samples of oocytes, early embryos or blastocysts from present or recent studies [7], [8]. Compared to humans, where large numbers of pre-implantation embryos are routinely examined, our data set is still small in size and might be influenced by statistical error.

Embryo mortality was estimated to reach up to 40% in pigs [22]. Screening of all chromosomes in porcine oocytes revealed approximately 10% of them to be aneuploid [7]. The incidence of aneuploidy increased in *in vivo* early porcine embryos to 14.3% [8], however decreased to 4.7% when examining higher stages of *in vivo* porcine embryos (blastocysts) in the current study. The frequency of true polyploidy in *in vivo* porcine embryos ranges between 0–6.7% [13], [21]–[26]. The combined data indicates that aneuploidy is not a major cause of aforementioned pregnancy loss in pigs.

## Materials and Methods

All animal work was conducted according to Act No 246/1992 Coll., on the protection of animals against cruelty under supervision of Central Commission for Animal Welfare, approval ID 018/2010.

### Embryo collection

For our experiments, 10 crossbreed LxCLW cycling gilts and 9 Prestice black pied cycling gilts (age, 8–10 months; weight approx. 130–150 kg) were used as embryo donors. The collection of embryos was performed according to a previously published protocol [8]. Briefly, estrous cycle was synchronized by Regumate (Intervet) over a 16-day period (daily 20 mg altrenogest per gilt). Four to 5 days after the treatment, the estrus onset was checked. Gilts were inseminated at the next naturally occuring estrus. Animals were slaughtered 5.5 days after insemination. The embryos were flushed from the uterus by phosphate buffered saline with the addition of 5% bovine fetal serum. The number of the blastocysts and late morulas was noted. Lower stages of embryos, if found, were not analyzed. Blastocysts from crossbreed LxCLW gilts were analyzed using CGH and blastocysts obtained from Prestice black pied gilts were used for FISH analysis. This was not desired, but was inevitable due to a change in pig breed at the local farm before realization of the FISH experiments.

### Whole genome amplification and comparative genomic hybridization

Blastocysts designated for CGH analysis of all chromosomes were washed in 0.01 N HCl in order to remove a *zona pellucida*, further washed in few droplets of sterile 10 mM Tris-HCl, pH 8.5 (Tris-HCl). Whole blastocyst were stored in 3 µl of Tris-HCl in a 200 µl PCR tube at −70°C until analyzed. Lysis of the whole blastocyst, WGA using Repli-g kit (Qiagen) and CGH was performed as previously described in [8] with only a minor modification: the use of Salmon testes DNA in a preparation step of the hybridization probe was omitted without any resulting deterioration in subsequent hybridizations. After the hybridization, metaphase chromosome spreads were examined using an Olympus BX 60 fluorescence microscope and analysis of captured images was performed with CGH-ISIS software (META systems, GmbH). For each CGH experiment, on average 5 good quality metaphase chromosome spreads were karyotyped and used for red∶green ratio calculation. A red∶green ratio of >1.25∶1 was indicative of chromosomal material gain, while ratio of <0.75∶1 indicated loss. Telomeric, centromeric and heterochromatic regions show variation among individuals due to dense distribution of repetitive sequences, so they were excluded from analysis.

### Fluorescent in situ hybridization

Locus-specific FISH was used to evaluate polyploidy in porcine blastocysts. Embryos were fixed on the slide using a Tween 20/HCl fixation technique [27] and interphase nuclei were analyzed using FISH probes for porcine chromosomes 1 and 10 directly labeled with Spectrum Green-dUTP and Spectrum Orange-dUTP (Abbott Molecular). The probe for chromosome 1 was prepared on the basis of DNA sequence data from the GenBank Nucleotide Sequence Database. The cosmid S0045 [28] was used as a probe for chromosome 10. The probe mixture and FISH procedure has been described elsewhere [13]. Also the scoring criteria for signal enumeration were applied according to this study.

The criteria for the determination of ploidy status were as follows:

Diploid nucleus: a nucleus was considered diploid if found present with 2 signals for one analyzed chromosome and with 2 or less signals for the second chromosome (the number of FISH signals were 2+2, 2+1, 2+0)

Haploid nucleus: a nucleus with 1+1 or 1+0 FISH signals

Triploid nucleus: a nucleus with 3+3 FISH signals only

Tetraploid nucleus: a nucleus with 4+4 or 4+3 FISH signals

Inconclusive nucleus: nuclei with 2+3 FISH signals were detected in 25 out of 4412 examined cells (0.0057%) and were scored as inconclusive. Other FISH signal combinations (e.g. 3+1, 4+2, 3+0) were seen very rarely and were also scored as inconclusive.
